# Supplementary material for: Time after ostomy surgery and type of treatment are associated with quality of life changes in colorectal cancer patients with colostomy
Source: PLoS One. 2020 Dec 3;15(12):e0239201. doi: 10.1371/journal.pone.0239201 (PMC7714142; doi:10.1371/journal.pone.0239201)
Supplement: S1 Table — * Mann-Whitney test for quantitative and chi-square for qualitative variables. (DOCX) [file pone.0239201.s001.docx]

**S1 Table.** Differences in the general characteristics of patients who remained in the study and those lost to follow-up

| **Variables** | **Participated T0-T1 (n=15)** | **Follow-up losses T0-T1 (n=26)** |  | **Participated T0-T2 (n=16)** | **Follow-up losses T0-T2 (n=25)** |  |
| --- | --- | --- | --- | --- | --- | --- |
|  | **% (n)**  **Mean (SD)** | | ***p-value** | **% (n)**  **Mean (SD)** | | ***p-value** |
| **Age** | 60.33 (10.39) | 62.19 (13.16) | 0.642 | 62.75 (7.78) | 60.72 (14.33) | 0.607 |
| **Gender** |  |  |  |  |  |  |
| Male | 46.7 (7) | 46.2 (12) | 0.975 | 37.5 (6) | 52.0 (13) | 0.376 |
| Female | 53.3 (8) | 53.8 (14) |  | 62.5 (10) | 48.0 (12) |  |
| **Staging** |  |  |  |  |  |  |
| I | 20.0 (3) | 23.1 (6) | 0.332 | 25.0 (4) | 20.0 (5) | 0.608 |
| II | 40.0 (6) | 7.7 (2) |  | 31.3 (5) | 12.0 (3) |  |
| III | 20.0 (3) | 34.6 (9) |  | 12.5 (2) | 40.0 (10) |  |
| IV | 0.0 (0) | 15.4 (4) |  | 6.3 (1) | 12.0 (3) |  |
| Pathological staging Y | 20.0 (3) | 11.5 (3) |  | 25.0 (4) | 8.0 (2) |  |
| Not specified or unknown | 0.0 (0) | 7.7 (2) |  | 0.0 (0) | 8.0 (2) |  |
| **Treatment** |  |  |  |  |  |  |
| Surgery only | 13.3 (2) | 15.4 (4) | 0.511 | 12.5 (2) | 16.0 (4) | 0.173 |
| Chemotherapy or radiotherapy | 26.7 (4) | 38.5 (10) |  | 18.8 (3) | 44.0 (11) |  |
| Chemoradiotherapy | 60.0 (9) | 46.6 (12) |  | 68.8 (11) | 40.0 (10) |  |
| **Nutritional status** | 24.54 (3.81) | 24.36 (4.43) | 0.812 | 24.26 (3.42) | 24.57 (4.65) | 0.720 |

* Mann-Whitney test for quantitative and chi-square for qualitative variables.
