# Supplementary material for: Time after ostomy surgery and type of treatment are associated with quality of life changes in colorectal cancer patients with colostomy
Source: PLoS One. 2020 Dec 3;15(12):e0239201. doi: 10.1371/journal.pone.0239201 (PMC7714142; doi:10.1371/journal.pone.0239201)
Supplement: S2 Table — *Mann-Whitney Test. (DOCX) [file pone.0239201.s002.docx]

**S2 Table.** EORTC-QLQ-C30 and EORTC-QLQ-CR29 differences between patients who remained in the study and those lost to follow-up

| **Quality of Life domains** | **Participated T0-T1 (n=15)** | **Follow-up losses T0-T1 (n=26)** |  | **Participated T0-T2 (n=16)** | **Follow-up losses T0-T2 (n=25)** |  |
| --- | --- | --- | --- | --- | --- | --- |
|  | **Median (min-max)** | | ***p-value** | **Median (min-max)** | | ***p-value** |
| **EORTC-QLQ-C30** |  |  |  |  |  |  |
| Global health status | 83.33 (33.33 - 100.00) | 75.00 (50.00 - 100.00) | 0.8364 | 79.17 (33.33 - 100.00) | 75.00 (50.00 - 100.00) | 0.6054 |
| Physical function | 80.00 (45.00 - 100.00) | 87.50 (50.00 - 100.00) | 0.6037 | 80.00 (50.00 - 100.00) | 90.00 (45.00 - 100.00) | 0.2247 |
| Role function | 83.33 (0.00 - 100.00) | 58.33 (0.00 - 100.00) | 0.2092 | 66.67 (0.00 - 100.00) | 66.67 (0.00 - 100.00) | 0.8594 |
| Emotional function | 66.67 (0.00 - 100.00) | 79.17 (8.33 - 100.00) | 0.7432 | 66.67 (0.00 - 100.00) | 83.33 (8.33 - 100.00) | 0.9248 |
| Cognitive function | 100.00 (33.33 - 100.00) | 100.00 (16.66 - 100.00) | 0.1268 | 100.00 (16.67 - 100.00) | 100.00 (50.00 - 100.00) | 0.0983 |
| Social function | 100.00 (0.00 - 100.00) | 100.00 (0.00 - 100.00) | 0.3705 | 100.00 (0.00 - 100.00) | 100.00 (0.00 - 100.00) | 0.8767 |
| Fatigue | 22.22 (0.00 - 100.00) | 27.78 (0.00 - 100.00) | 0.9454 | 33.33 (0.00 - 100.00) | 22.22 (0.00 - 100.00) | 0.4735 |
| Nausea and vomiting | 0.00 (0.00 - 100.00) | 0.00 (0.00 - 100.00) | 0.3961 | 0.00 (0.00 - 100.00) | 0.00 (0.00 - 100.00) | 0.5246 |
| Pain | 0.00 (0.00 - 100.00) | 16.67 (0.00 - 100.00) | 0.1954 | 0.00 (0.00 - 100.00) | 16.67 (0.00 - 100.00) | 0.1223 |
| Dyspnea | 0.00 (0.00 - 66.67) | 0.00 (0.00 - 100.00) | 0.4459 | 0.00 (0.00 - 66.67) | 0.00 (0.00 - 100.00) | 0.3842 |
| Insomnia | 33.33 (0.00 - 100.00) | 0.00 (0.00 - 100.00) | 0.7472 | 0.00 (0.00 - 100.00) | 33.33 (0.00 - 100.00) | 0.4606 |
| Appetite loss | 0.00 (0.00 - 100.00) | 33.33 (0.00 - 100.00) | 0.0723 | 0.00 (0.00 - 100.00) | 33.33 (0.00 - 100.00) | 0.1076 |
| Constipation | 0.00 (0.00 - 100.00) | 0.00 (0.00 - 100.00) | 0.5279 | 0.00 (0.00 - 100.00) | 0.00 (0.00 - 66.67) | 0.1074 |
| Diarrhea | 0.00 (0.00 - 100.00) | 0.00 (0.00 - 100.00) | 0.8756 | 0.00 (0.00 - 100.00) | 0.00 (0.00 - 66.67) | 0.0926 |
| Financial difficulties | 0.00 (0.00 - 100.00) | 16.67 (0.00 - 100.00) | 0.6560 | 0.00 (0.00 - 100.00) | 33.33 (0.00 - 100.00) | 0.5774 |
| **EORTC-QLQ-CR29** |  |  |  |  |  |  |
| Urinary frequency | 0.00 (0.00 - 100.00) | 0.00 (0.00 - 66.67) | 0.4225 | 0.00 (0.00 - 33.33) | 0.00 (0.00 - 100.00) | 0.0616 |
| Blood or mucus in stools | 0.00 (0.00 - 33.33) | 0.00 (0.00 - 50.00) | 0.0648 | 0.00 (0.00 - 33.33) | 0.00 (0.00 - 50.00) | 0.1474 |
| Stool frequency | 0.00 (0.00 - 83.33) | 0.00 (0.00 - 33.33) | 0.6741 | 0.00 (0.00 - 83.33) | 0.00 (0.00 - 33.33) | 0.6037 |
| Body image | 100.00 (0.00 - 100.00) | 88.89 (0.00 - 100.00) | 0.7180 | 100.00 (0.00 - 100.00) | 88.89 (0.00 - 100.00) | 0.2364 |
| Urinary incontinence | 0.00 (0.00 - 33.33) | 0.00 (0.00 - 0.00) | 0.0593 | 0.00 (0.00 - 33.33) | 0.00 (0.00 - 33.33) | 0.7473 |
| Dysuria | 0.00 (0.00 - 100.00) | 0.00 (0.00 - 66.67) | 0.4229 | 0.00 (0.00 - 100.00) | 0.00 (0.00 - 66.67) | 0.1635 |
| Abdominal pain | 0.00 (0.00 - 100.00) | 33.33 (0.00 - 100.00) | 0.4994 | 0.00 (0.00 - 100.00) | 33.33 (0.00 - 100.00) | 0.2643 |
| Buttock pain | 0.00 (0.00 - 100.00) | 0.00 (0.00 - 100.00) | 0.5739 | 0.00 (0.00 - 100.00) | 0.00 (0.00 - 100.00) | 1.0000 |
| Bloating | 0.00 (0.00 - 100.00) | 0.00 (0.00 - 100.00) | 0.8128 | 0.00 (0.00 - 100.00) | 0.00 (0.00 - 100.00) | 0.8030 |
| Dry mouth | 66.67 (0.00 - 100.00) | 50.00 (0.00 - 100.00) | 0.3218 | 50.00 (0.00 - 100.00) | 66.67 (0.00 - 100.00) | 0.9666 |
| Hair loss | 0.00 (0.00 - 100.00) | 0.00 (0.00 - 100.00) | 0.9722 | 0.00 (0.00 - 100.00) | 0.00 (0.00 - 66.67) | 0.9315 |
| Taste | 0.00 (0.00 - 100.00) | 0.00 (0.00 - 66.67) | 0.5309 | 0.00 (0.00 - 66.67) | 0.00 (0.00 - 100.00) | 0.3534 |
| Anxiety | 66.67 (0.00 - 100.00) | 66.67 (0.00 - 100.00) | 0.6612 | 33.33 (0.00 - 100.00) | 66.67 (0.00 - 100.00) | 0.2141 |
| Weight | 100.00 (0.00 - 100.00) | 100.00 (0.00 - 100.00) | 0.6877 | 100.00 (0.00 - 100.00) | 100.00 (0.00 - 100.00) | 0.1892 |
| Flatulence | 33.33 (0.00 - 100.00) | 33.33 (0.00 - 100.00) | 0.6489 | 16.67 (0.00 - 100.00) | 33.33 (0.00 - 100.00) | 0.5647 |
| Fecal incontinence | 0.00 (0.00 - 100.00) | 0.00 (0.00 - 100.00) | 0.0771 | 0.00 (0.00 - 100.00) | 0.00 (0.00 - 100.00) | 0.1757 |
| Sore skin | 0.00 (0.00 - 100.00) | 0.00 (0.00 - 100.00) | 0.8220 | 0.00 (0.00 - 100.00) | 0.00 (0.00 - 100.00) | 0.2362 |
| Embarrassment | 0.00 (0.00 - 100.00) | 0.00 (0.00 - 100.00) | 0.9372 | 0.00 (0.00 - 100.00) | 0.00 (0.00 - 100.00) | 0.8764 |
| Stoma care problems | 0.00 (0.00 - 100.00) | 0.00 (0.00 - 100.00) | 0.4375 | 0.00 (0.00 - 100.00) | 0.00 (0.00 - 100.00) | 0.7737 |
| Sexual interest (men) | 66.67 (0.00 - 66.67) | 0.00 (0.00 - 66.67) | 0.1272 | 0.00 (0.00 - 66.67) | 0.00 (0.00 - 66.67) | 0.8432 |
| Impotence | 0.00 (0.00 - 66.67) | 0.00 (0.00 - 100.00) | 0.8416 | 0.00 (0.00 - 100.00) | 0.00 (0.00 - 66.67) | 0.1467 |
| Sexual interest (women) | 0.00 (0.00 - 100.00) | 0.00 (0.00 - 33.33) | 0.4169 | 0.00 (0.00 - 66.67) | 0.00 (0.00 - 100.00) | 0.2595 |
| Dyspareunia | 0.00 (0.00 - 0.00) | 0.00 (0.00 - 0.00) | 1.0000 | 0.00 (0.00 - 0.00) | 0.00 (0.00 - 0.00) | 1.0000 |

*Mann-Whitney Test.
