# Supplementary material for: Time after ostomy surgery and type of treatment are associated with quality of life changes in colorectal cancer patients with colostomy
Source: PLoS One. 2020 Dec 3;15(12):e0239201. doi: 10.1371/journal.pone.0239201 (PMC7714142; doi:10.1371/journal.pone.0239201)
Supplement: S3 Table — *Mann-Whitney Test. (DOCX) [file pone.0239201.s003.docx]

**S3 Table.** EORTC-QLQ-C30 and EORTC-QLQ-CR29 differences between patients undergoing curative and palliative surgery and patients with temporary and permanent ostomy

| **Quality of Life domains** | **Curative (n=28)** | **Palliative (n=13)** |  | **Temporary (n=29)** | **Permanent (n=12)** |  |
| --- | --- | --- | --- | --- | --- | --- |
|  | **Median (min-max)** | | ***p-value** | **Median (min-max)** | | ***p-value** |
| **EORTC-QLQ-C30** |  |  |  |  |  |  |
| Global health status | 83.33 (41.67 – 100.00) | 75.00 (33.33 – 100.00) | 0.589 | 75.00 (41.67 – 100.00) | 83.33 (33.33 – 100.00) | 0.195 |
| Physical function | 87.5 (45.00 – 100.00) | 80.00 (65.00 – 100.00) | 0.923 | 90.00 (45.00 – 100.00) | 77.50 (55.00 – 100.00) | 0.300 |
| Role function | 50.00 (0.00 – 100.00) | 66.67 (16.67 – 100.00) | 0.128 | 50.00 (0.00 – 100.00) | 75.00 (16.67 – 100.00) | 0.314 |
| Emotional function | 75.00 (0.00 – 100.00) | 83.33 (3.33 - 100.00) | 0.709 | 75.00 (0.00 – 100.00) | 83.33 (8.33 – 100.00) | 0.419 |
| Cognitive function | 100.00 (16.67 – 100.00) | 100.00 (33.33 – 100.00) | 0.552 | 100.00 (16.67 – 100.00) | 91.67 (33.33 – 100.00) | 0.262 |
| Social function | 100.00 (0.00 – 100.00) | 66.67 (0.00 – 100.00) | 0.923 | 100.00 (0.00 – 100.00) | 91.67 (0.00 – 100.00) | 0.621 |
| Fatigue | 22.22 (0.00 – 100.00) | 33.33 (0.00 – 100.00) | 0.430 | 11.11 (0.00 – 100.00) | 33.33 (0.00 – 100.00) | 0.100 |
| Nausea and vomiting | 0.00 (0.00 – 100.00) | 0.00 (0.00 – 50.00) | 0.945 | 0.00 (0.00 – 100.00) | 0.00 (0.00 – 100.00) | 0.703 |
| Pain | 0.00 (0.00 – 100.00) | 33.33 (0.00 – 100.00) | 0.324 | 16.67 (0.00 – 100.00) | 25.00 (0.00 – 83.33) | 0.944 |
| Dyspnea | 0.00 (0.00 – 33.33) | 0.00 (0.00 – 100.00) | 0.750 | 0.00 (0.00 – 100.00) | 0.00 (0.00 – 66.67) | 0.810 |
| Insomnia | 16.67 (0.00 – 100.00) | 0.00 (0.00 – 100.00) | 0.945 | 0.00 (0.00 – 100.00) | 33.33 (0.00 – 100.00) | 0.832 |
| Appetite loss | 0.00 (0.00 – 100.00) | 0.00 (0.00 – 100.00) | 0.793 | 0.00 (0.00 – 100.00) | 0.00 (0.00 – 100.00) | 0.877 |
| Constipation | 0.00 (0.00 – 0.00) | 0.00 (0.00 – 100.00) | 0.121 | 0.00 (0.00 – 00.00) | 0.00 (0.00 – 100.00) | 0.100 |
| Diarrhea | 0.00 (0.00 – 100.00) | 0.00 (0.00 – 33.33) | 0.668 | 0.00 (0.00 – 100.00) | 0.00 (0.00 – 33.33) | 0.745 |
| Financial difficulties | 33.33 (0.00 – 100.00) | 0.00 (0.00 – 100.00) | 0.070 | 33.33 (0.00 – 100.00) | 0.00 (0.00 – 100.00) | **0.042** |
| **EORTC-QLQ-CR29** |  |  |  |  |  |  |
| Urinary frequency | 0.00 (0.00 – 100.00) | 0.00 (0.00 – 66.67) | 0.413 | 0.00 (0.00 – 100.00) | 0.00 (0.00 – 66.67) | 0.524 |
| Blood or mucus in stools | 0.00 (0.0 – 50.0) | 0.00 (0.00 – 50.00) | 0.901 | 0.00 (0.00 – 50.00) | 0.00 (0.00 – 16.67) | 0.506 |
| Stool frequency | 0.00 (0.00 – 83.33) | 0.00 (0.00 – 33.33) | 0.879 | 0.00 (0.00 – 83.33) | 0.00 (0.00 – 33.33) | 0.921 |
| Body image | 94.44 (0.00 – 100.00) | 88.89 (33.33 – 100.00) | 0.967 | 88.89 (0.00 – 100.00) | 100.00 (33.33 – 100.00) | 0.470 |
| Urinary incontinence | 0.00 (0.00 – 33.33) | 0.00 (0.00 – 33.33) | 0.836 | 0.00 (0.00 – 33.33) | 0.00 (0.00 – 33.33) | 0.810 |
| Dysuria | 0.00 (0.00 – 100.00) | 0.00 (0.00 – 66.67) | 0.750 | 0.00 (0.00 – 100.00) | 0.00 (0.00 – 33.33) | 0.767 |
| Abdominal pain | 0.00 (0.00 – 100.00) | 33.33 (0.00 – 100.00) | 0.090 | 0.00 (0.00 – 100.00) | 16.67 (0.00 – 100.00) | 0.832 |
| Buttock pain | 0.00 (0.00 – 100.00) | 0.00 (0.00 – 100.00) | 0.901 | 0.00 (0.00 – 100.00) | 0.00 (0.00 – 100.00) | 0.745 |
| Bloating | 0.0 (0.00 – 100.00) | 0.00 (0.00 – 100.00) | 0.628 | 0.00 (0.00 – 100.00) | 16.67 (0.00 – 100.00) | 0.621 |
| Dry mouth | 66.67 (0.00 – 100.00) | 33.33 (0.00 – 100.00) | 0.298 | 66.67 (0.00 – 100.00) | 50.00 (0.00 – 100.00) | 0.921 |
| Hair loss | 0.00 (0.00 – 100.00) | 0.00 (0.00 – 100.00) | 0.879 | 0.00 (0.00 – 100.00) | 0.00 (0.00 – 100.00) | 0.989 |
| Taste | 0.00 (0.00 – 100.00) | 0.00 (0.00 – 33.33) | 0.298 | 0.00 (0.00 – 100.00) | 0.00 (0.00 – 33.33) | 0.372 |
| Anxiety | 66.67 (0.00 – 100.00) | 66.67 (0.00 – 100.00) | 0.836 | 66.67 (0.00 – 100.00) | 66.67 (0.00 – 100.00) | 0.641 |
| Weight | 100.00 (0.00 – 100.00) | 100.00 (33.33 – 100.00) | 0.216 | 100.00 (0.00 – 100.00) | 100.00 (33.33 – 100.00) | 0.372 |
| Flatulence | 33.33 (0.00 – 100.00) | 33.33 (0.00 – 100.00) | 0.570 | 33.33 (0.00 – 100.00) | 16.67 (0.00 – 100.00) | 0.562 |
| Fecal incontinence | 0.00 (0.00 – 100.00) | 0.00 (0.00 – 100.00) | 0.552 | 0.00 (0.00 – 100.00) | 0.00 (0.00 – 33.33) | 0.088 |
| Sore skin | 0.00 (0.00 – 100.00) | 0.00 (0.00 – 100.00) | 0.668 | 0.00 (0.00 – 100.00) | 0.00 (0.00 – 33.33) | 0.328 |
| Embarrassment | 0.00 (0.00 – 100.00) | 33.33 (0.00 – 100.00) | 0.195 | 0.00 (0.00 – 100.00) | 33.33 (0.00 – 100.00) | 0.158 |
| Stoma care problems | 0.00 (0.00 – 100.00) | 0.00 (0.00 – 100.00) | 0.648 | 0.00 (0.00 – 100.00) | 0.00 (0.00 – 100.00) | 0.488 |
| Sexual interest (men) | 0.00 (0.00 – 66.67) | 0.00 (0.00 – 66.67) | 0.893 | 16.67 (0.00 – 66.67) | 0.00 (0.00 – 66.67) | 0.432 |
| Impotence | 0.00 (0.00 – 100.00) | 0.00 (0.00 – 66.67) | 0.893 | 0.00 (0.00 – 100.00) | 0.00 (0.00 – 66.67) | 0.536 |
| Sexual interest (women) | 0.00 (0.00 – 100.00) | 0.00 (0.00 – 33.33) | 0.714 | 0.00 (0.00 – 100.00) | 0.00 (0.00 – 33.33) | 1.000 |
| Dyspareunia | 0.00 (0.00 – 0.00) | 0.00 (0.00 – 100.00) | 1.000 | 0.00 (0.0 – 0.0) | 0.00 (0.00 – 0.00) | 1.000 |

*Mann-Whitney Test.
