# Supplementary material for: Time after ostomy surgery and type of treatment are associated with quality of life changes in colorectal cancer patients with colostomy
Source: PLoS One. 2020 Dec 3;15(12):e0239201. doi: 10.1371/journal.pone.0239201 (PMC7714142; doi:10.1371/journal.pone.0239201)
Supplement: Attachment — Submitted filename: Response to Reviewers.docx [file pone.0239201.s004.docx]

ANSWERS TO THE REVIEWER’S COMMENTS

(The responses are presented in red letter immediately below the reviewer’s comments. We realized that some Editor and Reviewer questions are similar, so we joined and answered together in the first part of this document).

**Editor comments**

**Question #1.** “This is an interesting study that addresses a key patient centered issue. In the revision it will be important to use patient centered language (e.g., "colostomized patient" should be rephrased to "patient living with a colostomy" etc.).”

**Comment to editor:** We appreciate and agree the Editor comment. We revised the manuscript and changed all sentences, as requested. Please see the new sentences lines 2, 47, 62, 100 – 101, 105, 164, 260, 270, 279, 365 and 373.

**Question #2.** “Reviewer #1 has raised an important concern that, I too, would like you to expand upon: the follow-up for this longitudinal study was very low, therefore issues to consider include: 1) Why were patients lost to follow-up; did some have ostomy reversal or for some other reason?”

**Comment to Editor and Reviewer#1:** We understand your concern. All steps involving this prospective study were carefully followed. Firstly, only patients who had undergone colostomy up to 2 months due to colorectal cancer were included (recent colostomies). Since this study is longitudinal, the patients were invited again in 3 and 6 months later, and these times established between the interviews (3 months) needed to be respected. If for any reason the patient took more than 3 months to attend the hospital/outpatient service, the interview was not considered in the present study. In addition, patients undergoing intestinal transit reconstruction during the study period were excluded. The main reasons for the loss of follow-up were (reported by patients): the distance from the health service, the number of questionnaires to assess the quality of life, the spent time in answering it, and distance and difficulty of locomotion to hospital/clinical service. In order to clarify the reasons for follow-up losses, we summarized the data below (please, see the flowchart and the question #4, similar to this).

Since all patients submitted to colostomy surgery were invited to participate, we do not perform the sample calculation before collect data (because we had invited all available individuals, i.e, the “universe”), and it is really happened. According data from the statistical sector of Clinical Hospital of Uberlandia, 348 colostomy surgeries were done between 2013 to April 2018 (mean of 63 per year – Please see the report below of statistical sector). So, a total of 65 patients obtained as the sample is in accordance with the service numbers. Although the complete collection period was 18 months, the entry allowed in the study was 12 months, the remaining 6 months was for the patients to complete T2.

Since all patients were invited and we did not perform a sample size calculation a priori as mentioned before, we performed post hoc effect size and observation power. After that, we showed these data and consider in manuscript only domains that showed enough power between times. We added new analysis about the losses in order to show the follow-up losses was not compromise our results. Please see the question #4.

As showed by the Figure below, all 65 patients underwent surgery at the hospital and/or followed-up at the outpatient clinic during the study period. Of these, 16 refused to participate. And 8 patients could not schedule, because they did not return to the hospital/outpatient service in the time foreseen for inclusion in the study. Thus, 41 patients met the inclusion criteria. After the first interview, we try scheduling and rescheduling attempts were made in the stipulated period of the study (3 months). However, 11 patients at T1 and 9 patients on T2 did not attend the return at the correct time, 3 and 6 months, respectively, were therefore not eligible within the stipulated time. In addition, 5 patients died, 3 lived in another city and 7 gave up. Among the reasons for withdrawal we can consider the time to answer the questionnaires and the displacement to the hospital service as main in the difficulty in the follow-up. Despite the loss of follow-up, there was no difference between the group that remained in the study of the one who gave up, neither in the general characteristics nor in the comparison of the quality of life domains.

**Patients invited for participations**

**(n=65)**

**Excluded (n=24)**

Did not accept participate in the study (n=16)

Patients could not schedule in the established time (n=8)

Total = 16

Total = 15

Total = 41

**T0**

**Ostomy time ≤2 months**

**T1**

**Ostomy time ≤2 months**

**Excluded (n=26)**

Death (n=5)

Interview was not done at the established time (n=11)

Lived in another city (n=3)

Patient quit (n=7)

**T2**

**Ostomy time ≤2 months**

**Excluded (n=25)**

Death (n=5)

Interview was not done at the established time (n=9).

Two patients no showed at T1 and attended the interview at T2

Lived in another city (n=3)

Ostomy reversal (n=1)

Patient quit (n=7)

Finally, we would like to add a comment about the sample sizes of the studies in the literature regarding the impact of ostomy in quality of life. Among the prospective studies about this context, most of them evaluated patients with and without an ostomy (Allal et al., 2005; Engel et al., 2003; Johansson et al., 2018; Yang et al., 2014; Yau et al., 2009). So, it is reasonable to think that the patient with ostomy could show poor quality of life when compared with patients without an ostomy. Besides that, some studies mixed patients with ileostomy and colostomy (Carlsson et al, 2010), or yet, they evaluated the impact of QOL on patients who underwent colectomy and not necessarily the ostomy (Theodoropoulos et al., 2013; Tsunoda et al., 2007; Camilleri-Brenan e Steele, 2001).

Considering the studies only with ostomies, the sample size ranged from 14 to 101 patients (considered the mixed population mentioned before). Only one study investigates exclusively patients with colostomy by CRC (n = 16, only 7 completed the study). Perhaps it happened due to the difficulty of find a higher sample (Ito et al., 2012). Therefore, despite the often-inevitable losses, we can consider the total obtained for our study as a considerable sample. Finally we also showed an enough power observation, as mentioned below and explained in manuscript. In other to clarify even more the manuscript we improve the sentences page 4, lines 74 to 87.

**References**

- Allal AS, Gervaz P, Gertsch P, Bernier J, Roth AD, Morel P, et al. Assessment of quality of life in patients with rectal cancer treated by preoperative radiotherapy: A longitudinal prospective study. Int J Radiat Oncol Biol Phys. 2005;61: 1129–1135. doi:10.1016/j.ijrobp.2004.07.726
- Camilleri-Brennan J, Steele RJC. Prospective analysis of quality of life and survival following mesorectal excision for rectal cancer. Br J Surg. 2001;88:1617–22. doi:10.1046/j.0007-1323.2001.01933.x
- Carlsson E, Berndtsson I, Hallén AM, Lindholm E, Persson E. Concerns and quality of life before surgery and during the recovery period in patients with rectal cancer and an ostomy. J Wound, Ostomy Cont Nurs. 2010;37: 654–661. doi:10.1097/WON.0b013e3181f90f0c
- Engel J, Kerr J, Schlesinger-Raab A, Eckel R, Sauer H, Hölzel D. Quality of Life in Rectal Cancer Patients. Ann Surg. 2003;238: 203–213. doi:10.1097/01.sla.0000080823.38569.b0
- Ito N, Ishiguro M, Uno M, Syunsyke K, Shimizu S, Obata R, et al. Prospective longitudinal evaluation of quality of life in patients with permanent colostomy after cu. J Wound, Ostomy, Cont Nurs Off Publ Wound, Ostomy Cont. 2012;39: 172. doi: 10.1097 / WON.0b013e3182456177
- Johansson AC, Brink E, Cliffordson C, Axelsson M. The function of fatigue and illness perceptions as mediators between self-efficacy and health-related quality of life during the first year after surgery in persons treated for colorectal cancer. J Clin Nurs. 2018;27: e1537–e1548. doi:10.1111/jocn.14300
- Theodoropoulos GE, Karantanos T, Stamopoulos P, Zografos G. Prospective evaluation of health-related quality of life after laparoscopic colectomy for cancer. Tech Coloproctol. 2013;17: 27–38. doi:10.1007/s10151-012-0869-7
- Tsunoda A, Nakao K, Hiratsuka K, Tsunoda Y, Kusano M. Prospective analysis of quality of life in the first year after colorectal cancer surgery. Acta Oncol (Madr). 2007;46: 77–82. doi:10.1080/02841860600847053
- Yang X, Li Q, Zhao H, Li J, Duan J. Quality of life in rectal cancer patients with permanent colostomy in Xi’an. African Health Sciences. 2014;14: 28–36.
- Yau T, Watkins D, Cunningham D, Barbachano Y, Chau I, Chong G. Longitudinal assessment of quality of life in rectal cancer patients with or without stomas following primary resection. Dis Colon Rectum. 2009;52: 669–677. doi:10.1007/DCR.0b013e31819eb970

**Question #3.** “2) Were there any factors measured at baseline that were predictive of loss to follow up?”

**Comment to Reviewer:** We understood and agree with the Reviewer’s concern. As mentioned in question #2, all patients submitted to colostomy surgery were invited to participate. Even with a loss of 36.9% (24/65), we have 63% of all surgery done in the study period. In addition, we performed the size effect for each time to observe the power observation a posteriori. As a result, many quality of life domains showed enough power observation. Finally, we added new analysis about the losses in order to show the follow-up losses was not compromise our results. Please see the question #4 and the new results in the Supplementary Material.

**Question #4.** “And 3) How does your statistical model handle this issue of missing data? It is plausible to think that patients with poor quality of life might withdraw from the study, which would impact your findings if only patients with good quality of life remained in the analytic sample. A robust evaluation and acknowledgement of this issue will strengthen this paper. We look forward to receiving the revised manuscript.”

**Comment to Reviewer:** We appreciate the important Reviewer comment. In order to understand if the patients with poor quality of life might withdraw from the study, we performed new analysis (please see the tables below). The S1 Table shows the ANOVA and chi-square tests in order to understand if there were difference in the general characteristics of patients who remained in the study and lost follow-up. As we can observe, there were not significant differences for age, sex, tumor staging, types of treatment and nutrition status between who remained and withdraw of the study.

In addition, the S2 Table shows the differences for EORTC-QLQ-C30 and EORTC-QLQ-CR29 between patients who remained in the study and lost follow-up (Mann-Whitney Test). In the same way as S1 Table, the quality of life domains were not showed differences, among patients who left the study of those who remained. In addition, it is important to emphasize the method of statistical analysis Generalized Estimation Equations (GEE) is the most appropriate method to proceed longitudinal analyses which the estimative of models considering the patient is his own control, being compared to himself over time (Wang et al 2013).

Therefore, we add the S1 and S2 tables as supplementary material. In order to clarify this point, we add also sentences in the Methods, Results and Discussion sections (Page 8, lines 175 to 183; page 15, lines 217 to 228; and page 25, lines 367 to 369).

Reference:

- Wang M, Kong L, Li Z, Zhang L. Covariance estimators for generalized estimating equations (GEE) in longitudinal analysis with small samples. Stat Med. 2016;35(10):1706-1721. doi:10.1002/sim.6817.

**S1 Table.** Differences in the general characteristics of patients who remained in the study and those lost to follow-up

| **Variables** | Participated T0-T1 (n=15) | Follow-up losses T0-T1 (n=26) |  | Participated T0-T2 (n=16) | Follow-up losses T0-T2 (n=25) |  |
| --- | --- | --- | --- | --- | --- | --- |
|  | % (n)  Mean (SD) | | *p-value | % (n)  Mean (SD) | | *p-value |
| **Age** | 60.33 (10.39) | 62.19 (13.16) | 0.642 | 62.75 (7.78) | 60.72 (14.33) | 0.607 |
| **Gender** |  |  |  |  |  |  |
| Male | 46.7 (7) | 46.2 (12) | 0.975 | 37.5 (6) | 52.0 (13) | 0.376 |
| Female | 53.3 (8) | 53.8 (14) |  | 62.5 (10) | 48.0 (12) |  |
| **Staging** |  |  |  |  |  |  |
| I | 20.0 (3) | 23.1 (6) | 0.332 | 25.0 (4) | 20.0 (5) | 0.608 |
| II | 40.0 (6) | 7.7 (2) |  | 31.3 (5) | 12.0 (3) |  |
| III | 20.0 (3) | 34.6 (9) |  | 12.5 (2) | 40.0 (10) |  |
| IV | 0.0 (0) | 15.4 (4) |  | 6.3 (1) | 12.0 (3) |  |
| Pathological staging Y | 20.0 (3) | 11.5 (3) |  | 25.0 (4) | 8.0 (2) |  |
| Not specified or unknown | 0.0 (0) | 7.7 (2) |  | 0.0 (0) | 8.0 (2) |  |
| **Treatment** |  |  |  |  |  |  |
| Surgery only | 13.3 (2) | 15.4 (4) | 0.511 | 12.5 (2) | 16.0 (4) | 0.173 |
| Chemotherapy or radiotherapy | 26.7 (4) | 38.5 (10) |  | 18.8 (3) | 44.0 (11) |  |
| Chemoradiotherapy | 60.0 (9) | 46.6 (12) |  | 68.8 (11) | 40.0 (10) |  |
| **Nutritional status** | 24.54 (3.81) | 24.36 (4.43) | 0.812 | 24.26 (3.42) | 24.57 (4.65) | 0.720 |

* Mann-Whitney Test for quantitative and chi-square variables for qualitative variables.

**Table SB.** EORTC-QLQ-C30 and EORTC-QLQ-CR29 differences between patients who remained in the study and those lost to follow-up

| **Quality of Life domains** | Participated T0-T1 (n=15) | Follow-up losses T0-T1 (n=26) |  | Participated T0-T2 (n=16) | Follow-up losses T0-T2 (n=25) |  |
| --- | --- | --- | --- | --- | --- | --- |
|  | **Median (min-max)** | | ****p-value*** | **Median (min-max)** | | ****p-value*** |
| **EORTC-QLQ-C30** |  |  |  |  |  |  |
| Global health status | 83.33 (33.33 - 100.00) | 75.00 (50.00 - 100.00) | 0.8364 | 79.17 (33.33 - 100.00) | 75.00 (50.00 - 100.00) | 0.6054 |
| Physical function | 80.00 (45.00 - 100.00) | 87.50 (50.00 - 100.00) | 0.6037 | 80.00 (50.00 - 100.00) | 90.00 (45.00 - 100.00) | 0.2247 |
| Role function | 83.33 (0.00 - 100.00) | 58.33 (0.00 - 100.00) | 0.2092 | 66.67 (0.00 - 100.00) | 66.67 (0.00 - 100.00) | 0.8594 |
| Emotional function | 66.67 (0.00 - 100.00) | 79.17 (8.33 - 100.00) | 0.7432 | 66.67 (0.00 - 100.00) | 83.33 (8.33 - 100.00) | 0.9248 |
| Cognitive function | 100.00 (33.33 - 100.00) | 100.00 (16.66 - 100.00) | 0.1268 | 100.00 (16.67 - 100.00) | 100.00 (50.00 - 100.00) | 0.0983 |
| Social function | 100.00 (0.00 - 100.00) | 100.00 (0.00 - 100.00) | 0.3705 | 100.00 (0.00 - 100.00) | 100.00 (0.00 - 100.00) | 0.8767 |
| Fatigue | 22.22 (0.00 - 100.00) | 27.78 (0.00 - 100.00) | 0.9454 | 33.33 (0.00 - 100.00) | 22.22 (0.00 - 100.00) | 0.4735 |
| Nausea and vomiting | 0.00 (0.00 - 100.00) | 0.00 (0.00 - 100.00) | 0.3961 | 0.00 (0.00 - 100.00) | 0.00 (0.00 - 100.00) | 0.5246 |
| Pain | 0.00 (0.00 - 100.00) | 16.67 (0.00 - 100.00) | 0.1954 | 0.00 (0.00 - 100.00) | 16.67 (0.00 - 100.00) | 0.1223 |
| Dyspnea | 0.00 (0.00 - 66.67) | 0.00 (0.00 - 100.00) | 0.4459 | 0.00 (0.00 - 66.67) | 0.00 (0.00 - 100.00) | 0.3842 |
| Insomnia | 33.33 (0.00 - 100.00) | 0.00 (0.00 - 100.00) | 0.7472 | 0.00 (0.00 - 100.00) | 33.33 (0.00 - 100.00) | 0.4606 |
| Appetite loss | 0.00 (0.00 - 100.00) | 33.33 (0.00 - 100.00) | 0.0723 | 0.00 (0.00 - 100.00) | 33.33 (0.00 - 100.00) | 0.1076 |
| Constipation | 0.00 (0.00 - 100.00) | 0.00 (0.00 - 100.00) | 0.5279 | 0.00 (0.00 - 100.00) | 0.00 (0.00 - 66.67) | 0.1074 |
| Diarrhea | 0.00 (0.00 - 100.00) | 0.00 (0.00 - 100.00) | 0.8756 | 0.00 (0.00 - 100.00) | 0.00 (0.00 - 66.67) | 0.0926 |
| Financial difficulties | 0.00 (0.00 - 100.00) | 16.67 (0.00 - 100.00) | 0.6560 | 0.00 (0.00 - 100.00) | 33.33 (0.00 - 100.00) | 0.5774 |
| **EORTC-QLQ-CR29** |  |  |  |  |  |  |
| Urinary frequency | 0.00 (0.00 - 100.00) | 0.00 (0.00 - 66.67) | 0.4225 | 0.00 (0.00 - 33.33) | 0.00 (0.00 - 100.00) | 0.0616 |
| Blood or mucus in stools | 0.00 (0.00 - 33.33) | 0.00 (0.00 - 50.00) | 0.0648 | 0.00 (0.00 - 33.33) | 0.00 (0.00 - 50.00) | 0.1474 |
| Stool frequency | 0.00 (0.00 - 83.33) | 0.00 (0.00 - 33.33) | 0.6741 | 0.00 (0.00 - 83.33) | 0.00 (0.00 - 33.33) | 0.6037 |
| Body image | 100.00 (0.00 - 100.00) | 88.89 (0.00 - 100.00) | 0.7180 | 100.00 (0.00 - 100.00) | 88.89 (0.00 - 100.00) | 0.2364 |
| Urinary incontinence | 0.00 (0.00 - 33.33) | 0.00 (0.00 - 0.00) | 0.0593 | 0.00 (0.00 - 33.33) | 0.00 (0.00 - 33.33) | 0.7473 |
| Dysuria | 0.00 (0.00 - 100.00) | 0.00 (0.00 - 66.67) | 0.4229 | 0.00 (0.00 - 100.00) | 0.00 (0.00 - 66.67) | 0.1635 |
| Abdominal pain | 0.00 (0.00 - 100.00) | 33.33 (0.00 - 100.00) | 0.4994 | 0.00 (0.00 - 100.00) | 33.33 (0.00 - 100.00) | 0.2643 |
| Buttock pain | 0.00 (0.00 - 100.00) | 0.00 (0.00 - 100.00) | 0.5739 | 0.00 (0.00 - 100.00) | 0.00 (0.00 - 100.00) | 1.0000 |
| Bloating | 0.00 (0.00 - 100.00) | 0.00 (0.00 - 100.00) | 0.8128 | 0.00 (0.00 - 100.00) | 0.00 (0.00 - 100.00) | 0.8030 |
| Dry mouth | 66.67 (0.00 - 100.00) | 50.00 (0.00 - 100.00) | 0.3218 | 50.00 (0.00 - 100.00) | 66.67 (0.00 - 100.00) | 0.9666 |
| Hair loss | 0.00 (0.00 - 100.00) | 0.00 (0.00 - 100.00) | 0.9722 | 0.00 (0.00 - 100.00) | 0.00 (0.00 - 66.67) | 0.9315 |
| Taste | 0.00 (0.00 - 100.00) | 0.00 (0.00 - 66.67) | 0.5309 | 0.00 (0.00 - 66.67) | 0.00 (0.00 - 100.00) | 0.3534 |
| Anxiety | 66.67 (0.00 - 100.00) | 66.67 (0.00 - 100.00) | 0.6612 | 33.33 (0.00 - 100.00) | 66.67 (0.00 - 100.00) | 0.2141 |
| Weight | 100.00 (0.00 - 100.00) | 100.00 (0.00 - 100.00) | 0.6877 | 100.00 (0.00 - 100.00) | 100.00 (0.00 - 100.00) | 0.1892 |
| Flatulence | 33.33 (0.00 - 100.00) | 33.33 (0.00 - 100.00) | 0.6489 | 16.67 (0.00 - 100.00) | 33.33 (0.00 - 100.00) | 0.5647 |
| Fecal incontinence | 0.00 (0.00 - 100.00) | 0.00 (0.00 - 100.00) | 0.0771 | 0.00 (0.00 - 100.00) | 0.00 (0.00 - 100.00) | 0.1757 |
| Sore skin | 0.00 (0.00 - 100.00) | 0.00 (0.00 - 100.00) | 0.8220 | 0.00 (0.00 - 100.00) | 0.00 (0.00 - 100.00) | 0.2362 |
| Embarrassment | 0.00 (0.00 - 100.00) | 0.00 (0.00 - 100.00) | 0.9372 | 0.00 (0.00 - 100.00) | 0.00 (0.00 - 100.00) | 0.8764 |
| Stoma care problems | 0.00 (0.00 - 100.00) | 0.00 (0.00 - 100.00) | 0.4375 | 0.00 (0.00 - 100.00) | 0.00 (0.00 - 100.00) | 0.7737 |
| Sexual interest (men) | 66.67 (0.00 - 66.67) | 0.00 (0.00 - 66.67) | 0.1272 | 0.00 (0.00 - 66.67) | 0.00 (0.00 - 66.67) | 0.8432 |
| Impotence | 0.00 (0.00 - 66.67) | 0.00 (0.00 - 100.00) | 0.8416 | 0.00 (0.00 - 100.00) | 0.00 (0.00 - 66.67) | 0.1467 |
| Sexual interest (women) | 0.00 (0.00 - 100.00) | 0.00 (0.00 - 33.33) | 0.4169 | 0.00 (0.00 - 66.67) | 0.00 (0.00 - 100.00) | 0.2595 |
| Dyspareunia | 0.00 (0.00 - 0.00) | 0.00 (0.00 - 0.00) | 1.0000 | 0.00 (0.00 - 0.00) | 0.00 (0.00 - 0.00) | 1.0000 |

*Mann-Whitney Test.

**Question #5.** “Please ensure that your manuscript meets PLOS ONE's style requirements, including those for file naming. The PLOS ONE style templates can be found at <https://journals.plos.org/plosone/s/file?id=wjVg/PLOSOne_formatting_sample_main_body.pdf> and <https://journals.plos.org/plosone/s/file?id=ba62/PLOSOne_formatting_sample_title_authors_affiliations.pdf> “

**Comment to Editor:** Thank for the Editor comment. We revised all style requirements and update the format of our manuscript, as requested.

**Question #6.** “Please include a caption for figure 2.”

**Comment to Editor:** We added the caption in the Figure 2. Please see the page 20, line 248 to 250.

**Reviewer #1**

**Question #7.** “I am very honored that the authors and your journal have asked me to review this article. This article is a good study. They used three methods to analyze the recovery of quality of life in patients with colorectal cancer with ostomy, providing guidance for the clinical health treatment team. “Can the "using a specific instrument" on line 282 be specific, such as the method or tool used?”

**Comment to Reviewer:** We appreciate the Reviewer comment. Regarding the question about the term "using a specific instrument" we referred to the specific tool used, i.e., the EORTC-QLQ-CR29 questionnaire. We modified it to make the text more clear. Please see the new sentences page, 21 line 280.

**Reviewer #2:**

**Question #8.** “Overall: nice prospective study with some interesting findings. The English language needs substantial revision with regards to grammar and phrasings.”

**Comment to Reviewer:** We agree with the reviewer. The English language was revised by a proofreading service. Please, find enclosed the revised manuscript and the English review certificate below.

**Question #9.** “Furthermore, both introduction and discussion sections could benefit from some trimming. References should be scrutinized again; several of the references just mentioned once could be referenced numerous times.”

**Comment to Reviewer:** We agree with the Reviewer. We revised all Introduction and Discussion sections, as requeted. In addition, we added the references in several sentences, when appropriate. Please see the new sentences in page 4, lines 74 to 87; page 21, lines 270 to 278; page 22, lines 291 to 299 and lines 311 to 313; and page 23, lines 316 to 319.

**Question #10.** “Some references I cannot examine owing to language barrier (e.g. 17, 18, 19, 35, 45) and one I cannot find in neither google scholar, pubmed nor embase (#23, Salles et al). I believe the high number of references could be reduced by for example using most recent literature, that of better quality and with those with populations resembling that of the present study.”

**Comment to Reviewer:** We agree with the Reviewer and would like to apologize for this. We revised all references and we maintain just those in English language. We also reviewed all the Introduction and Discussion sections where we found the citations. As requested, we altered the references in several sentences, when appropriate. We only maintain the Pan American Health Organization reference (before #35, now #29) in the Methods section (page 6, line 142) because it is the reference for the elderly. Anyway, we improve the references to become easier to find it. Please see the new sentences in page 4, line 73; page 4, lines 74 to 79, page 21, lines 272 and 290; page 22, line 313; page 23, lines 321 to 324 and line 331.

**Question #11.** “There is a substantial drop out /loss to follow-up from T0 to T1 and T2. Reason for this should be explained better along with potential significant differences between patients answering T1 and T2 and those who are lost to follow-up.”

**Comment to Reviewer:** We agree and understand the Reviewer’s concern. We described and showed all information and details about these points in #1, #2, #3, and #4 questions. In addition, we also showed the new analyzes that we include in the manuscript as supplementary material. Please, see the explanations mentioned before.

**Question #12.** “Especially since it is difficult to work out whether this study concerns curative treatment only. If any patients undergo palliative treatment or have recurrence this (curative vs. palliative treatment and recurrence) should be presented in table 1 as it potentially affects HRQoL.”

**Comment to the reviewer:** We agree and understand the Reviewer's concern. We performed a revision in all clinical records. We had 28 patients undergo curative and 13 undergo palliative intent surgery. We added this information in Table 1, as requested, and we add new sentences in the Methods and Results sections, page 5, lines 105 to 106; and page 9, line 204.

**Introduction:**

**Question #13.** “Could be trimmed in order to improve presentation of the study objective. First 2/3 seeks to justify the study but a more clear presentation of the problem and study objective would improve the section.”

**Comment to Reviewer:** We agree with the reviewer and we made some modifications in this section as requested. Please see the new sentences lines 74 to 94.

**Question #14.** “Numerous studies exist on HRQoL in ostomized CRC patients using cancer specific intruments (the EORTC QLQ-C30 and -CR29: Verweij et al 2017, Brauman&Müller et al 2016, Kasparek et al 2011, Mols et al 2014, Zajac 2008, Mahjoubi et al 2012 among others) so this sentence should be rephrased.”

**Comment to Reviewer:** We understand the Reviewer’s concern. We agree that many studies investigated the HRQoL in ostomized CRC patients using cancer specific instruments. Some manuscripts cited by reviewer were also cited in our study (cross-sectional studies). However, there are scarce relevant studies with prospective design developed with the exclusive sample of ostomized by CRC. It is important to highlight there are some cross-sectional studies (Verweij et al 2017, Mahjoubi et al 2012), or study that includes groups with and without ostomies (Kasparek et al 2011, Mols et al 2014). It is important to note the symptoms are different between colostomy/ileostomy, or, with/without colostomy for example. Brauman & Müller et al 2016 also performed a cross-sectional study, in which included many diseases that resulting in an ostomy. So, these studies not included only patients with colostomy due colorectal cancer. Regarding Zajac et al 2008 cross-sectional study with 50 rectal cancer patients, besides not specifying which time of ostomy was considered as "the early post-surgery follow-up period", this study did not show any statistical test neither clinical difference observation. So, it is really scarce relevant prospective studies in the literature about this discussed point. Besides that, in the longitudinal studies, the patients are their own controls over time. Because of this, we did not cite all these studies in our manuscript. In order to clarify this part and attend the doubt of the reviewer, we rewrite some sentences. A prospective study with a specific sample may clarify the cause-effect relationship between ostomy time and quality of life, different from the other published manuscripts before. In order to achieve the Reviewer expectations, the phrase mentioned was improved in the text. Please see the new sentences in lines 74 to 79.

**Methods:**

**Question #15.** “Inclusion/esclusion criteria:It is unclear to me if this is study includes patients undergoing curative therapy alone or if some patients undergo palliative treatment. This is very important when evaluating HRQoL.”

**Comment to Reviewer:** We understand the Reviewer's concern. As previously mentioned in question #11, after reviewing all clinical records, we had 28 patients undergo potential curative and 13 undergo palliative intent surgeries. Please see the new sentences lines 105 to 106. We would like to point out that the records of patients who underwent curative surgery were made according to intention of primary treatment. We can only say if the colorectal cancer treatment was curative after 5 years of follow-up (Godhi et al., 2017).

Reference:

- Godhi S, Godhi A, Bhat R, Saluja S. Colorectal Cancer: Postoperative Follow-up and Surveillance. Indian J Surg. 2017;79(3):234-237. doi:10.1007/s12262-017-1610-6.

**Question #16.** “It is also unclear to me if some patients have chemotherapy during some (but not all) of time points.”

**Comment to Reviewer:** We agree and understand the Reviewer’s concern. The standardized treatment protocol is neoadjuvant chemoradiotherapy for patients with rectal cancer and surgery for patients with colon cancer. When it is not possible to total tumor removal, the patients were submitted to an adjuvant chemotherapy. Of the 41 patients included in the study, 6 did not undergo chemoradiotherapy, as showed in chart below. We added a sentence in the Methods to clarify section. Please see the new sentences, page 6, lines 126 to 128.

| **Treatment** | **Before surgery** | **After surgery** | **Before and after surgery** |
| --- | --- | --- | --- |
|  | **n** | | |
| Chemotherapy | 0 | 9 | 1 |
| Radiotherapy | 4 | 0 | 0 |
| Chemoradiotherapy | 15 | 3 | 3 |

**Question #17.** “The phrase "Negative clinically changes" can be confusing at times. 'Improvement' and 'worsening' for example is easier understood.”

**Comment to Reviewer:** We appreciate and agree with the Reviewer comment. We changed the terms the "Negative clinically changes" only when it did not change its meaning. Please see the new sentences in page 2, lines 31 and 38; page 8, line 191; and page 23, line 327.

**Question #18.** “CR-29 is not exclusively for stoma patients. It is for colorectal cancer patients. This should be changed.”

**Comment to Reviewer:** We agree with the reviewer. In fact, the CR29 was not exclusive for stoma patients, but for the colorectal cancer. This sentence was modified. Please see the new sentences lines 150 to 152.

**Question #19.** “I am not familiar with all of the applied statistical methods, and cannot assess whether appropriate methods are used. There are some repetitions in this sections, and it could be shortened considerably.”

**Comment to Reviewer:** We agreed with the reviewer. We improve this part of the manuscript. Please see the new sentences in the Methods section, page 8, lines 164 to 183.

**Question #20.** “The last part of the section is especially hard to understand.”

**Comment to Reviewer:** We agree with the Reviewer. The minimum clinically important it is a little confusing and we improve this part in manuscript (page 8, lines 184 to 192). Initially, the minimum clinically important difference was defined as a minimum amount of change reported by the patient capable of to capture the magnitude of the improvement or worsening and the value that patients attribute to the change (Cook, 2008; McGlothlin and Lewis, 2014). Any amount of change greater than the limit of the minimum clinically important difference is consider “significant” or important. Many manuscripts valorize this point because of their practical point of view (Cella et al, 2002; Singer et al, 2019). Therefore, this difference was used to complement the study, as statistical analyses may be significant based on numerical values and at the same time have no clinical importance for the quality of life of patients (Copay et al, 2007). Following other studies (Sloan et al, 2006; Singer et al, 2019), for the calculation of cutoff of the evaluated population, the standard deviation of the domain score was multiplied by the size of the effect (0.5). As in the present studies are many domains, we consider the overall score of QOL (SD = 18.45), resulting in 9.23. Therefore, patients who worsened ≥ ± 9 points on the time scales were considered clinically important.

References:

- Cella D, Eton DT, Lai JS, Peterman AH, Merkel DE. Combining anchor and distribution-based methods to derive minimal clinically important differences on the Functional Assessment of Cancer Therapy (FACT) anemia and fatigue scales. J Pain Symptom Manage. 2002;24(6):547-561. doi:10.1016/s0885-3924(02)00529-8
- Cook CE. Clinimetrics Corner: The Minimal Clinically Important Change Score (MCID): A Necessary Pretense. *J Man Manip Ther*. 2008;16(4):E82-E83. doi:10.1179/jmt.2008.16.4.82E
- Copay AG, Subach BR, Glassman SD, Polly DW Jr, Schuler TC. Understanding the minimum clinically important difference: a review of concepts and methods. *Spine J*. 2007;7(5):541-546. doi:10.1016/j.spinee.2007.01.008
- McGlothlin AE, Lewis RJ. Minimal Clinically Important Difference: Defining What Really Matters to Patients. *JAMA*. 2014;312(13):1342–1343. doi:10.1001/jama.2014.13128
- Sloan JA, Frost MH, Berzon R, Dueck A, Guyatt G, Moinpour C, et al. The clinical significance of quality of life assessments in oncology: A summary for clinicians. Support Care Cancer. 2006;14: 988–998. doi:10.1007/s00520-006-0085-y
- Singer S, Amdal CD, Hammerlid E, Tomaszewska IM, Castro Silva J, Mehanna H, et al. International validation of the revised European Organisation for Research and Treatment of Cancer Head and Neck Cancer Module, the EORTC QLQ-HN43: Phase IV. Head Neck. 2019; 1–13. doi:10.1002/hed.25609

**Results:**

**Question #21.** “Table 1: As mentioned I would like to see: curative vs. palliative treatment and recurrence in this table. As well as temporary/permanent colostomy distributions.”

**Comment to Reviewer:** We appreciate this important Reviewer comment. We add these data, as reviewer requested (we showed this information also in questions #12 and #15). After revised all clinical records, we observe 28 and 13 curative and palliative intent surgeries, respectively. We also had 29 temporary and 12 permanent colostomies. Only patients with colostomy in interviews were included in our study (inclusion criteria). Please see the new sentences page 9, line 204.

**Question #22.** “Tables 2A and 2B: QoL data are skewed but by convention EORTC data are most often presented as means and SD but not necessarily median and IQR. Adhering to this could improve overview of these tables. Or else the reason for presenting both means AND medians should be explained. Significance test should of course consider the skewness of data (non parametric tests)”

**Comment to Reviewer:** We understand the Reviewer’s concern. In fact it is common to see the mean (with their standardized deviation) and median (interquartiles ranges or min-max values). We chose to present the data in both ways since the measures represent the central tendency, complementing the reader's understanding. Since the tests are related to the nature of the non-parametric data, we perform a comparison of medians. Some studies have also shown median and IQR (Peng et al., 2011; Carlsson et al., 2010; Bloemen et al., 2009). However, if the reviewer finds this change extremely important, we are not opposed to represent the data only the mean and standard deviation.

References:

- Peng J, Shi D, Goodman KA, et al. Early results of quality of life for curatively treated rectal cancers in Chinese patients with EORTC QLQ-CR29. *Radiat Oncol*. 2011;6:93. Published 2011 Aug 12. doi:10.1186/1748-717X-6-93
- Carlsson E, Berndtsson I, Hallén AM, Lindholm E, Persson E. Concerns and quality of life before surgery and during the recovery period in patients with rectal cancer and an ostomy. *J Wound Ostomy Continence Nurs*. 2010;37(6):654-661. doi:10.1097/WON.0b013e3181f90f0c
- Bloemen JG, Visschers RG, Truin W, Beets GL, Konsten JL. Long-term quality of life in patients with rectal cancer: association with severe postoperative complications and presence of a stoma. *Dis Colon Rectum*. 2009;52(7):1251-1258. doi:10.1007/DCR.0b013e3181a74322

**Question #23.** “As mentioned comparison of disease characteristic of patients who answer T1 and T2 and those who are lost to follow-up should be presented. There are a substantial risk of bias here that must be addressed. If you know reasons who patients dropped out these should be presented.”

**Comment to Reviewer:** We understand and agree with the Reviewer’s concern. We detailed this context in the #2, #3 and #4 questions. Please see the explanation mentioned before. In order to clarify this point in the manuscript, we added this information as a limitation of the present study please see the new sentences in the Discussion section, page 24, lines 354 to 364.

**Discussion:**

**Question #24.** “This section could also be trimmed considerable focusing on the research questions covered by this study. Some sentences are completely abundant. It is highly debatable if it makes any sense discussing differences that are non significant even if clinically relevant (since they were significant). And drawing conclusions based on this should be done with more caution.”

**Comment to Reviewer:** We appreciate and agree the Reviewer comment. We reduce some sentences. However, we would like to add a little comment about domains that did not present significant results in the statistical analysis and it should be considered as clinically important. This is a different practical point of view and maybe deserves to be explored, as other studies (Cella et al, 2002; Maringwa et al, 2011; Zeng et al, 2012).

According to the Manual of the European Organisation for Research and Treatment of Cancer (EORTC) "the fact that a change is statistically significant does not necessarily imply that it also has clinical significance". The ‘Minimal Clinically Important Difference’ are grouped into anchor-based and distribution-based interpretations. That is why we perform both types of analysis. Please, see the new text in lines 317 to 330.

References:

- Cella D, Eton DT, Lai JS, Peterman AH, Merkel DE. Combining anchor and distribution-based methods to derive minimal clinically important differences on the Functional Assessment of Cancer Therapy (FACT) anemia and fatigue scales. J Pain Symptom Manage. 2002;24(6):547-561. doi:10.1016/s0885-3924(02)00529-8
- Fayers PM, Aaronson NK, Bjordal K, Groenvold M, Curran D, Bottomley, A. The EORTC QLQ-C30 scoring manual. European Organisation for Research and Treatment of Cancer. Brussels, 2001.
- Maringwa, J.T., Quinten, C., King, M. et al. Minimal important differences for interpreting health-related quality of life scores from the EORTC QLQ-C30 in lung cancer patients participating in randomized controlled trials. Support Care Cancer 19, 1753–1760 (2011). https://doi.org/10.1007/s00520-010-1016-5
- Zeng, L., Chow, E., Zhang, L. et al. An international prospective study establishing minimal clinically important differences in the EORTC QLQ-BM22 and QLQ-C30 in cancer patients with bone metastases. Support Care Cancer 20, 3307–3313 (2012). https://doi.org/10.1007/s00520-012-1484-x

**Question #25.** “The discussion of limitations and strengths should be more exhaustive. Using interviews rather than self-completion should be explained. The igh number of loss to follow up should be addressed and if all patients are not treated with curative intent this should be stated. This should also the timing of oncological treatment and how this may have affected outcomes. You cannot write the study has a higher response rate with a drop-out rate of 26/41=63%. It seems you are referring to missing items which will be small in interview-studies.”

**Comment to Reviewer:** We agree with the Reviewer. We revise the limitations and strengths and detail it more, as requested. Please, see the new text in lines 348 to 369. We hope to live up the Reviewer's expectations.
